# Global Assessment of Pediatric Patient Safety Tool for identifying safety incidents in pediatric patients

**DOI:** 10.1590/1984-0462/2023/41/2022076

**Published:** 2023-05-15

**Authors:** Marilise Borges Brandão, Ana Paula Hermann, Mônica Nunes Lima

**Affiliations:** a Universidade Federal do Paraná, Curitiba, PR, Brazil.

**Keywords:** Risk management, Adverse events, Gestão de risco, Eventos adversos

## Abstract

**Objective:**

The aim of this study was to evaluate the accuracy of the Global Assessment of Pediatric Patient Safety (GAPPS) in order to identify patient safety incidents with patient harm or adverse events (AEs).

**Methods:**

This is a cross-sectional, retrospective study of 240 records of hospitalized patients of both genders under 18 years of age, systematically and randomly selecting 10 charts of patients that meet the GAPPS criteria every 15 days from the 4,041 records of 2017.

**Results:**

The prevalence of AEs was 12.5%, i.e., detected in 30 out of 240 medical records. In total, 53 AEs and 63 harm were recorded, of which 53 (84.1%) were temporary and 43 AE (68.2%) were definitely or probably preventable. The presence of at least one trigger in a medical chart revealed 13 times greater chance of the occurrence of an AE, with sensitivity index of 48.5%, specificity of 100%, and accuracy of 86.5%.

**Conclusion:**

GAPPS was effective in detecting patient safety incidents with harm or AE.

## RESUMO

**Objetivo:**

Avaliar a acurácia da *Global Assessment of Pediatric Patient Safety* (GAPPS) para a identificação de incidente de segurança do paciente com dano ou evento adverso (EA).

**Métodos:**

Estudo transversal, retrospectivo, de 240 prontuários de pacientes internados com idade inferior a 18 anos e de ambos os sexos, com seleção randomizada sistemática de dez prontuários por quinzena, entre 4.041 prontuários que cumpriram os critérios da GAPPS no ano de 2017.

**Resultados:**

A prevalência de incidente de segurança do paciente foi de 12,5%, ou seja, ele foi detectado em 30 dos 240 prontuários. Foram registrados 53 EA e 63 danos ao paciente, dos quais 53 (84,1%) temporários. Quarenta e três EA (68,2%) foram considerados como definitiva ou provavelmente preveníveis. A presença de pelo menos um gatilho no prontuário apresentou índice de sensibilidade de 48,5%, especificidade de 100%, acurácia de 86,5% e chance 13 vezes maior de ocorrência de um EA.

**Conclusões:**

A GAPPS foi efetiva para a detecção de incidente de segurança do paciente com dano ou evento adverso.

## INTRODUCTION

A patient safety incident (PSI) with harm, also called adverse event (AE), is an event or circumstance that results in unnecessary harm to a patient under care, which is not an expected result of the progression of its underlying disease, resulting in impairment of the patient’s structure or organic functions. It is also defined as any harmful social, psychological, or physical effect on a patient, such as disease, injury, suffering, disability, or death.^1,2^ Hospitalized children are also susceptible to AEs, with estimated frequency of 11.1 AEs per 100 pediatric patients in wards, 74 AEs per 100 patients admitted to neonatal intensive care units (NICUs), and 203 AEs per 100 patients admitted to pediatric ICUs.^3-5^ The AEs have been recognized as a major cause of morbidity and mortality in this population, with an estimated death rate of above 4,000 per year in the USA, despite the efforts to promote patient safety.^6^

The most frequently used strategies for detection of AE are voluntary notification of PSI — anonymous, confidential, manually, or electronically written via software — and review of patients’ medical records using trigger tools. Triggers represent signs, symptoms, or situations that are assumed to be indicative of the occurrence of an AE.^7^ Over the past two decades, trigger tools have been developed as active, reliable, systematic, global, structured strategies, and of acceptable cost, for surveillance, detection, and monitoring of the occurrence of AE, upon review of a sample of randomly selected medical records.^8^ Researchers at the Center of Excellence and Measuring of Pediatric Quality, USA, developed a trigger tool in 2016, funded by the Agency for Healthcare Research and Quality (AHRQ), Division of General Pediatrics, at Boston Children’s Hospital, USA, specifically for pediatrics — the Global Assessment of Pediatric Patient Safety (GAPPS).^4^ The aim was to target the specifics of child care to identify AE. GAPPS was developed in several stages, and at the end, 37 triggers were selected to build the tool. For each trigger, an associated memo was written to standardize its definition and guide the use of the tool. The GAPPS Manual of Operations was released in February 2016 by the Center of Excellence for Pediatric Quality Measurement.^9^ In Brazil, GAPPS has not yet been translated, validated, or used as a systematic and global tool to identify AE in pediatrics. As a result, this study was conducted to evaluate the accuracy of GAPPS for the identification of AE in a public, federal, and teaching hospital in the south of Brazil.

## METHOD

This is a cross-sectional, retrospective study in a teaching hospital in southern Brazil of 240 medical records of hospitalized patients of both genders under 18 years of age, systematically and randomly selecting 10 charts of patients that meet the GAPPS criteria, every 15 days from the 4,041 records of 2017. Criteria were patients hospitalized for over 24 h, in pediatric or general beds, with any outcome (discharge, transfer, or death). Cases of admission for psychiatric treatment or rehabilitation and admission to day hospital were excluded, as well as newborns that remained in joint accommodation. Data collection was manual and performed from March to October 2019. A formulary was built with the final 37 triggers selected and proposed by GAPPS (27 triggers for manual research in physical records and 10 more triggers that could be detected in an automated way, if there are patients’ electronic records) for data collection, distributed in six categories:

1. Medications/fluids,
2. Care environment,
3. Health care-related infections,
4. Transfer and outcomes,
5. Surgical, and
6. Intensive care^3^ (Table 1).

**Table 1.** Global Assessment of Pediatric Patient Safety — modules and triggers.

Medication module/fluids – MF
MF1 Serum creatinine duplication
MF2 Use of nephrotoxin (e.g., aminoglycosides, cyclosporine, tacrolimus, and vancomycin) and increasing creatinine (Cr)
MF3 Hepatotoxic medicinal products and elevated liver enzymes
MF4 Hypoglycemia (<2 mmol/L egg 40 mg/dL)
MF5 Constipation related to opiates with intermittent laxative
MF6 Administration of naloxone (Narcan)
MF7 Warfarin triggers: RNI >6
MF7 High drug levels (antiepileptics): phenytoin (>30 μg/mL)
MF8 High drug levels (antiepileptics): oxcarbazepine (>45 μg/mL)
MF9 Bilirubin >25 mg/dL (<28 days of age)
MF10 Administration of flumazenil
MF11 Abrupt discontinuation of medication
Care environment module – AC
AC1 Infiltrations: infiltration/extravasation or phlebitis documentation
AC2 Pressure injury record (≥stage 2)
AC3 Embolus/thrombosis record
AC4 Infiltrations: administration of hyaluronidase
AC5 Health-associated infections: positive *Clostridioides difficile* test
AC6 Patient fall
Iras module – MI
MI1 Health-associated infections: positive blood culture (only after 48 h after admission)
MI2 Health-associated infections: positive urine culture (only after 48 h after admission)
MI3 Health-related infections: positive respiratory or gastrointestinal (GI) viral infection (only after 48 h after admission)
MI4 Oral vancomycin
MI5 Surgical site inf ection
Transfers and outcomes module – TD
TD1 Unplanned hospital readmission within 30 days
TD2 Cardiorespiratory arrest or rapid response team activation
TD3 All deaths of hospitalized patients
Surgical module – MC
MC1 Hemoglobin (Hgb) or hematocrit (Hct) drop of >25% in <24 h
MC2 Mechanical ventilation for a period of >48 h postoperatively
MC3 Return to the operating room
MC4 Operative time >6 h (noncardiac patients)
MC5 Intraoperative epinephrine, norepinephrine, or phenylephrine (noncardiac patients)
MC6 Change in procedure
Intensive care module – IT
TI1 Endotracheal extubation failure (reintubation within 24 h of planned extubation)
TI2 Use of racemic adrenaline (mechanically ventilated patients in the last 24 h)
TI3 ICU readmission within 24 h after discharge/transfer
TI4 Unplanned endotracheal extubation
TI5 Transfer to a higher level of care

The research was conducted in three stages, according to the instructions in the GAPPS Operating Manual:

1. Primary review of the 240 selected medical records was performed by the lead medical researcher for detection of the 37 triggers of the GAPPS tool, suspicion of AE, and collection of demographic data, diagnoses, and procedures performed at admission;
2. Secondary review, presenting cases with suspected AE to a pediatric intensive care specialist, to discuss and define the presence of an AE, plus the therapeutic interventions applied as a result of the AE; and
3. Consensus meeting held with presentation of cases with confirmed AE to a team of patient safety specialists at the institution, composed by a doctor, a nurse, and a pharmacist with experience in trigger tools to define which were and how many AE occurred, their severity, preventability, and category of care.

The measures of central tendency and dispersion are expressed in means and standard deviation for the continuous variables of a symmetric distribution and in medians and interquartile intervals for the variables of an asymmetric distribution. The estimated difference of categorical variables was performed by the Pearson’s/Yates’s chi-square test. Sensitivity, specificity, positive and negative predictive value, false-positive and false-negative indices, and accuracy were estimated, considering the AE as the gold standard and the triggers as factors. The odds ratio (OR) was calculated to estimate the association of GAPPS triggers with the occurrence of AEs. The sample was constituted according to the GAPPS guidelines, and for all tests, the significance level of 5% was considered (Statistica — StaSoft^®^). The study was approved by the Institution’s Ethics Committee on Research in Human Beings (Certificate of Presentation of Ethical Appreciation no. 89672018.6.0000.0096, opinion number 2.697.381) in 2019.

## RESULTS

The sample used was composed of 240 medical records, of which 115 (47.9%) were male and 125 (52.1%) were female patients, mostly hospitalized in a pediatric unit (80.0%) (Table 2). In the primary review, 122 triggers were detected in 76 (31.7%) medical records. In 43 medical records, 69 AEs were suspected, 54 (78.3%) of them with triggers and 15 (21.7%) without them, showing a higher frequency of triggers among patients with AEs.

**Table 2.** Patient characteristics.

Characteristics	n (%)
Gender	
Male	115 (47.9)
Female	125 (52.1)
Skin color	
White	204 (85.0)
Brown	35 (14.6)
Yellow	1 (0.4)
Pediatrics hospitalizations	192 (80.0)
Clinical	154 (80.2)
Surgical	38 (19.8)
Adult area hospitalizations	48 (20.0)
Clinical	17 (35.4)
Surgical	18 (37.5)
Obstetrics and gynecology	13 (27.1)
Type of hospitalization	
Elective	75 (31.2)
Emergency	165 (68.8)

n=240.

In the secondary review, 69 suspected AEs were analyzed, with 55 (79.7%) confirmed AEs and 32 (58.2%) had 47 associated triggers. The reviewers agreed on 66 harms to patients resulting from the 55 AEs recognized, and of these, 56 (84.8%) needed therapeutic intervention in 2 h following the AE. The most common interventions were new vascular access, reintubation, new gastric/intestinal probing, and injury suturing.

The consensus meeting defined that 53 AEs and 63 harms were on 30 medical records. Regarding severity, in 53 (84.1%) cases, the harm was temporary; in 1 (1.6%) case, the harm was permanent; in 8 (12.7%) cases, there was a need life support intervention; and in 1 (1.6%) case, death occurred. Notably, 43 (68.2%) harms were considered definitive or probably preventable. The main triggers detected were in the module medications/fluids with 42 (34.4%), followed by intensive care with 40 (32.7%), transfers and outcomes with 21 (17.2%), surgery with 14 (11.5%), and health care-related infections with 5 (4.0%). There were no triggers detected in the module of care and environment. The most frequent categories of error were procedure (31.7%), respiratory therapy (26.3%), infection related to health care (18.9%), infusion and nutritional therapy (10.8%), and medication (5.4%) (Table 3).

**Table 3.** Frequency of error category of preventable adverse events by type of error.

Error category	Number of harms (%)	Type of error
Medication	2 (5.4)	Missed dose
Procedure	5 (15.5)	Process error
	6 (16.2)	Difficulty or technical failure
Respiratory therapy	5 (15.5)	Necessary care not performed
	3 (8.1)	Inadequate patient preparation
	1 (2.7)	Process error
Infusion therapy	4 (10.8)	Necessary care not performed
Nutritional therapy	4 (10.8)	Necessary care not performed
Health care-related infection	2 (5.4)	Ventilator associated pneumonia
	2 (5.4)	Surgical site infection
	2 (5.4)	Sepsis/bacteremia not related to central catheter
	1 (2.7)	Bloodstream related to catheter

n=37.

GAPPS triggers showed 100% specificity, but with low sensitivity and good accuracy. The presence of at least one trigger per medical record showed a sensitivity index of 48.5%, specificity of 100%, accuracy of 86.5%, and 13 times greater chance of occurrence of an AE. The triggers that led to a higher level of care and abrupt interruption of medication were associated with 3 and 4 times greater chance of occurrence of AE, respectively (Table 4). However, there was no association between these triggers or at least one GAPPS trigger with the severity of the harm caused by AE. Almost all (97.2%) cases received discharge from the hospital to their home, and 2 (0.8%) patients died during hospitalization. The number of patients-days was 1,566, with 33 AEs per 1,000 patients-day, 22 AEs per 100 hospitalizations, 27 preventable AEs per 1,000 patients-day, and 17 preventable AEs per 100 hospitalizations.

**Table 4.** Global Assessment of Pediatric Patient Safety triggers and the chance of adverse event.

Trigger	OR	95%CI	p-value
Transference	3.45	1.60–7.42	<0.01
Discontinuation of medication	3.77	1.59–8.92	<0.01
At least one trigger	12.89	6.63–25.05	<0.001

OR: odds ratio; 95%CI: 95% confidence interval.

## DISCUSSION

In the present study, the prevalence of AE was 12.5%, i.e., detected in 30 of the 240 medical records. In total, 53 AEs and 63 harms were recorded. Of this, 53 (84.1%) harms were temporary. Notably, 43 (68.2%) AEs were considered definitive or probably preventable. The presence of at least one trigger in the medical records presented a sensitivity index of 48.5%, specificity of 100%, accuracy of 86.5%, and 13 times greater chance of occurrence of an AE. The detection of PSI is a fundamental approach for the promotion of safe health care.

The trend of health services is to employ different strategies for the identification of AEs, with voluntary notification being the most commonly used. However, limitations ranging from underreporting and the fragility of the nonpunitive safety culture to the low awareness rate for patient safety actions point to the need to establish active search mechanisms for AE in a health organization committed to its prevention. The complete review of medical records searching for AEs, although effective, is costly and time-consuming. In the past two decades, trigger tools for the detection of AEs have emerged, which have been shown to be able to detect severe AEs up to 10 times more when compared to other established methods. Initially developed for adult patients, trigger tools were later applied to or adapted for use in pediatrics. In 2016, the GAPPS was developed specifically for application in pediatrics, when triggers were selected, which then began to make up the tool after submission to a panel of specialists.^3^

In 2017, Stroupe et al.^10^ observed that GAPPS detected four times more AEs than voluntary notification in a pediatric hospital, and in 2018, Stockwell et al.^11^ conducted in 16 academic and nonacademic hospitals in 4 regions of the United States and detected the occurrence of 19.1 AEs per 1,000 patients-day and 9.5 preventable AEs per 1,000 patients-day. On average, teaching hospitals had higher rates of AEs.^12^ Matlow et al.,^13^ in a review of pediatric patient records, using the Canadian Pediatrics Trigger Tool, demonstrated that 15% of hospitalized children suffered AE from health care. Other studies with hospitalized children using different methods and/or tools for detecting AE reported the occurrence of 11.1 AEs per 100 hospitalized,^3-5,14^ which points to the understanding that GAPPS is a useful tool for the detection of AE in pediatric patients and can contribute to the monitoring of its occurrence as indicators of patient safety.

The present study indicated that some triggers detected or the presence of at least one trigger proposed by GAPPS did contribute significantly, as described, to the detection of AE, suggesting that a more detailed examination of the medical record should be performed. The U.K. Pediatric Trigger Tool (UKPTT) also showed that the triggers varied in their ability to lead to the identification of an AE and that some triggers, despite being frequently identified, presented very low positive predictive values.^15^ It also showed that in 85% of the cases, the harm was temporary; in 1.5% of the cases, an intervention, initial hospitalization, or prolongation was necessary; in another 1.5%, the AE contributed to or caused permanent harm to the patient; and in 1.5%, the AE contributed to or led to the patient’s death. In 2014, Chapman et al.,^15^ using UKPTT in 25 hospitals in the United Kingdom, observed similar results, with temporary harm in 92.2% of cases, permanent harm in 4.3% of cases, and death in 1.7% of cases.^15^ This study also pointed out that 54–82% of AEs could have been avoided, as also indicated by the Health Quality & Safety Commission of New Zealand in 2016.^16^

GAPPS presented an accuracy of 86.5%, with AE detection of 12.5%, with 63 AE, mostly (84.1%) temporary and 68.5% of them preventable. The presence of at least one trigger was associated with a change 13-fold greater of an AE.

The objectives were to apply the GAPPS and verify its accuracy for the detection of AE in pediatrics but limited to a period of 12 months. The use of this tool needs a learning curve for the professionals involved in the different stages of its application, which is a limiting factor in this study. The application of GAPPS in a systematic manner, on an annual basis, in a greater number and more diverse pediatric hospitals in different countries may contribute to improve the tool, demonstrate its cost-effectiveness and real impact on patients’ safety, and allow comparisons between different hospitals.

## Data Availability

The database that originated the article is available with the corresponding author.
